# Novel Plant Extract Ameliorates Metabolic Disorder through Activation of Brown Adipose Tissue in High-Fat Diet-Induced Obese Mice

**DOI:** 10.3390/ijms23169295

**Published:** 2022-08-18

**Authors:** Ji-Won Kim, Young-Mo Yang, Eun-Young Kwon, Ji-Young Choi

**Affiliations:** 1 Department of Food Sciences and Nutrition, Kyungpook National University, Daegu 41566, Korea; 2 Department of Pharmacy, College of Pharmacy, Chosun University, Gwangju 61452, Korea; 3 Department of Food and Nutrition, College of Natural Science and Public Health and Safety, Chosun University, Gwangju 61452, Korea

**Keywords:** *Phlomis umbrosa* Turcz. (Labiatae) root, high-fat diet, hepatic steatosis, browning, thermogenesis, energy expenditure

## Abstract

Obesity is characterized by excessive body fat accumulation due to unbalanced energy intake and expenditure. Potential therapeutic targets for anti-obesity include the inhibition of white adipose tissue (WAT) hypertrophy and hyperplasia and the activation of brown adipose tissue (BAT). Not only the activation of BAT but also the browning of WAT have gained increasing attention in research fields as an alternative method in the prevention and treatment of obesity. Here, we investigated possible mechanisms underlying the anti-obesity effect of *Phlomis umbrosa* Turcz. root ethanol extract (PUE) in an obesogenic animal model. PUE treatment can reduce diet-induced obesity and modulate obesity-associated metabolic disorders, including insulin resistance, hepatic steatosis, and inflammation. In the liver, PUE improved hepatic steatosis by suppressing hepatic lipogenesis and lipid absorption while increasing biliary sterol excretion and hepatic fatty acid oxidation compared to the high-fat group. Moreover, PUE increased energy expenditure and regulated fecal lipid excretion, leading to reduced body weight gain. In particular, PUE remarkably activated the browning of subWAT via upregulation of the browning-related protein and gene expression and promoted BAT activation. In conclusion, these findings provide the potential therapeutic usefulness into the effects of PUE in the treatment of obesity and metabolic disorders. Furthermore, it suggests that PUE treatment can regulate energy metabolism via activating BAT and browning subWAT.

## 1. Introduction

According to the World Health Organization (WHO), there were roughly 1.9 billion overweight adults and 650 million obese adults as of 2016. In 2017, more than 4 million people died because of being overweight or obese. In addition, 39 million children under the age of 5 were overweight or obese in 2020 [1]. Obesity is a condition in which metabolic disorders are caused by the accumulation of fat in the body. It is strongly associated with the development of hypertension, type 2 diabetes mellitus (T2DM), dyslipidemia, and non-alcoholic fatty liver disease (NAFLD). If energy intake is relatively higher than consumption, the amount and volume of adipose tissue increases, with an eventual corresponding increase in mass [2]. The main role of adipose tissue is to store energy in the body and secrete adipokines as an active endocrine organ [3]. However, dysregulation of lipid metabolism causes an increase in adipose tissue inflow to the liver and destroys the balance of lipid metabolism, thereby resulting in obesity-induced fatty liver. This damages hepatocytes, which accelerate inflammatory responses by increasing the expression of various inflammatory cytokines or by causing an abnormal regulation of adipokines [4]. Adiponectin is an adipocyte-specific adipokine with anti-arteriosclerotic and anti-inflammatory properties that improves insulin resistance by inhibiting hepatic gluconeogenesis. Adiponectin promotes energy consumption and fatty acid oxidation (FAO) through AMP-activated protein kinase (AMPK) activation and hence is involved in enhancing insulin sensitivity [5]. In the process of de novo lipogenesis, FFAs are produced and moved through the blood to the liver tissue. A high concentration of FFAs suppresses the action of insulin in hepatocytes and raises blood glucose [6]. Chronic diseases that result from obesity, such as diabetes, cardiovascular disease, and cancer, have been revealed as the major causes of death globally. Therefore, it is important to prevent and treat obesity in order to suppress this increase in chronic diseases [1]. 

Brown adipose tissue (BAT) produces heat, which increases energy expenditure. The browning of subcutaneous WAT (subWAT) also plays an important role in thermogenesis [7,8]. ‘Browning’ is a phenomenon in which WAT becomes brown, and these brown-like adipocytes found in WAT are referred to as brite/beige adipocytes [9]. When WAT is exposed to low temperatures, lipolysis and thermogenesis increase in the tissue, which is similar to the function of BAT. It is necessary to have sufficient uncoupling protein 1 (UCP1) in that process; UCP1 is thus an important biomarker for the browning mechanism [10]. This process has been mainly studied in epididymal WAT (epiWAT) in the past; it has also been found that subWAT has a high expression of browning-related markers [11]. Consequently, BAT activation and browning is regarded as a new target for obesity treatment [12].

*Phlomis umbrosa* Turcz. (Labiatae) (*P. umbrosa*) has been used in traditional medicine for a long time in Korea and Asia. To date, the root and leaf extracts of *P. umbrosa* have been found to have various positive effects, such as treating female menopause [13], child bone development and growth [14], osteoporosis [15], anti-inflammation, and antioxidation [16]. However, the anti-obesity effects of *P. umbrosa* have not yet been identified. 

Therefore, we investigated the inhibitory effect of *P. umbrosa* root extract on fat formation in 3T3-L1 adipocytes in a preliminary study. This study was undertaken to evaluate the anti-obesity effects of *P. umbrosa* root ethanol extract (PUE) on adiposity, dyslipidemia, and hepatic steatosis by upregulating energy expenditure via BAT activation and WAT browning in diet-induced obese (DIO) C57BL/6J mice.

## 2. Results

### 2.1. PUE Treatment Decreased Lipid Accumulation While Promoting Expression of Browning-Specific Genes in 3T3-L1 Adipocytes

**Figure 1:**
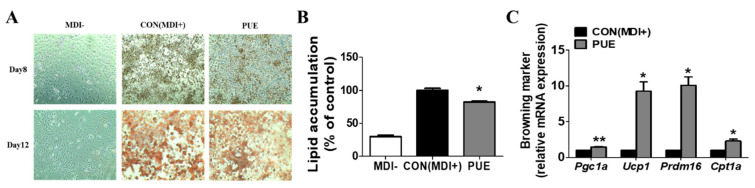
Effect of *P. umbrosa* root extract on lipid accumulation and browning-specific gene expression in 3T3-L1 adipocytes. (**A**) Oil red O staining (×200); (**B**) Lipid accumulation (%); (**C**) Browning-specific gene expression. (**B**,**C**) Data are presented as means ± SEM. Significant differences between CON (MDI+) and PUE are indicated by * *p* < 0.05; ** *p* < 0.01. MDI-, MDI-untreated cells; CON (MDI+), MDI-treated cells; PUE, MDI + *P. umbrosa* root extract, 100 μg/mL. MDI, methylisobutylxanthine, dexamethasone, and insulin; *Pgc1a*, Peroxisome proliferator-activated receptor gamma co-activator 1-alpha; *Ucp1*, Uncoupling protein 1; *Prdm16*, PR domain containing 16; *Cpt1a*, Carnitine palmitoyl-CoA transferase 1-alpha.

Figure 1A shows the results of Oil Red O staining after 8 and 12 days of adipocyte differentiation in 3T3-L1 cells treated with the differentiation drugs methylisobutylxanthine, dexamethasone, and insulin (MDI) and PUE (100 μg/mL). It was found that PUE reduced the accumulation of lipid droplets (Figure 1A,B). Moreover, PUE supplementation significantly increased the expression of browning markers (*Pgc1a*, *Ucp1*, *Prdm16,* and *Cpt1a*) compared to that in the CON (MDI+) group (Figure 1C).

### 2.2. PUE Improved Body, Organ, and Adipose Tissue Weights by Regulating Lipid Metabolism

**Figure 2:**
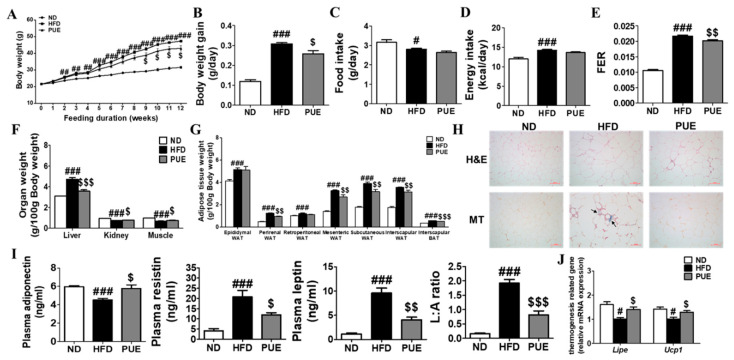
Effect of *P. umbrosa* root extract supplement on body and fat weights, adipose tissue morphology, adipokines, and mRNA expression in DIO mice. (**A**) Body weight; (**B**) Body weight gain; (**C**) Food intake; (**D**) Energy intake; (**E**) FER; (**F**) Organ weight; (**G**) Adipose tissue weight; (**H**) Epididymal WAT morphology (×200); (**I**) Plasma adipokines; (**J**) Epididymal WAT gene expression. (**A**–**G**,**I**,**J**) Data are presented as means ± SEM.; ND, normal diet (AIN-93G); HFD, high-fat diet (60 kcal% fat); PUE, HFD + *P. umbrosa* root extract (1% *w*/*w*). Significant differences between HFD and ND are indicated: ^#^ *p* < 0.05; ^##^ *p* < 0.01; ^###^ *p* < 0.001. Significant differences between HFD and PUE are indicated: ^$^ *p* < 0.05; ^$$^ *p* < 0.01; ^$$$^ *p* < 0.001. (**H**) Arrow indicates fibrosis-positive staining (blue). FER, food efficiency ratio; WAT, white adipose tissue; H&E, hematoxylin and eosin staining; MT, Masson’s trichrome staining; L:A ratio, leptin:adiponectin.

The body weight of the high-fat diet (HFD) group animals was markedly higher from week 2 compared to that of the normal diet (ND) group, whereas that of the PUE group significantly decreased after week 9 compared to the HFD cohort (Figure 2A). The average daily body weight gain (BWG) for 12 weeks (g/day) in the PUE group was significantly lower than that in the HFD group (Figure 2B). There were no significant differences in food and energy intake between the HFD and PUE groups (Figure 2C,D). This led to a significantly lower food efficiency ratio (FER) in the PUE group than in the HFD group (Figure 2E). The liver weights of the HFD group were significantly higher than those of the ND animals, while those of the PUE group were significantly lower than that of the HFD group. In addition, kidney and muscle weights were significantly higher in the PUE group than in the HFD group (Figure 2F). The HFD group exhibited significantly increased adipose tissue weight and adipocyte size compared with the ND cohort. However, PUE supplementation significantly decreased the perirenal, mesenteric, subcutaneous, and interscapular WAT and BAT weights and adipocyte size. WAT collagen accumulation was assessed by Masson’s trichrome (MT) staining, and fibrosis (stained blue) clearly progressed in the HFD group, but not in the ND and PUE groups (Figure 2G,H). Plasma adiponectin levels were significantly higher in the PUE group than in the HFD group. The plasma leptin, resistin, and leptin: adiponectin (L:A) ratio values in the HFD group were significantly increased compared to those in the ND group; their values in the PUE supplementation group were markedly lower than those in the HFD group (Figure 2I). The mRNA expression of *Lipe* in epiWAT was markedly increased in the ND and PUE groups compared to that in the HFD group. In addition, PUE significantly upregulated the mRNA expression of thermogenic genes such as *Ucp1* in epiWAT (Figure 2J).

### 2.3. PUE Increased Energy Expenditure and Fecal Lipid Excretion Levels

**Figure 3:**
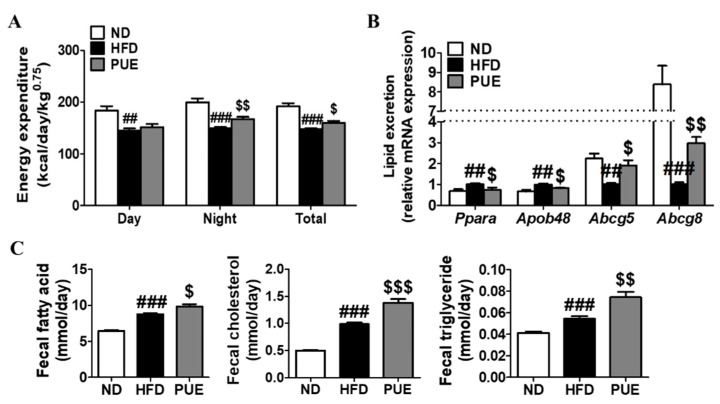
Effect of *P. umbrosa* root extract supplement on energy expenditure, small intestine gene expression related to lipid excretion, and fecal lipid content in DIO mice. (**A**) Energy expenditure; (**B**) Small intestine gene expression; (**C**) Fecal lipid contents. (**A**–**C**) Data are presented as means ± SEM.; ND, normal diet (AIN-93G); HFD, high-fat diet (60 kcal% fat); PUE, HFD + *P. umbrosa* root extract (1% *w*/*w*). Significant differences between HFD and ND are indicated by ^##^ *p* < 0.01; ^###^ *p* < 0.001. Significant differences between HFD and PUE are indicated by ^$^ *p* < 0.05; ^$$^ *p* < 0.01; ^$$$^ *p* < 0.001.

The energy expenditure of the HFD group was markedly lower than that of the ND group during day and night periods. In contrast, PUE supplementation significantly augmented energy expenditure at night compared to the HFD group. The metabolic rate measurements of energy expenditure per day were significantly higher in the PUE group than in the HFD group (Figure 3A). Changes in the mRNA expression of genes related to cholesterol transport and lipid excretion in the small intestine were assessed (Figure 3B). PUE supplementation resulted in significant downregulation of the lipid transporter genes *Ppara* and *Apob48* compared to the HFD group. Moreover, the mRNA levels of *Abcg5* and *Abcg8* in the small intestine of PUE-exposed mice were significantly higher than those in HFD-fed mice (Figure 3B). Fecal lipid excretion was significantly higher in HFD-fed mice than in ND-fed mice (*p* < 0.001), and PUE supplementation significantly increased fecal lipid excretion compared with the other diet groups (Figure 3C).

### 2.4. PUE Supplementation Improved Plasma Lipid Levels

The HFD group showed significantly higher plasma triglyceride (TG) and high-density lipoprotein cholesterol (HDL-C) levels than the ND group. These lipid levels were not affected by PUE supplementation, whereas plasma total cholesterol (TC), phospholipid (PL), and non-HDL-C levels were increased in HFD-fed animals and were significantly lower in the PUE group than in the HFD group. In addition, the PUE group showed a significantly increased apo A-1/apo B ratio compared to the HFD group (Table 1).

### 2.5. PUE Downregulated Hepatic Glucose Enzyme Activities and Attenuated Insulin Resistance

**Figure 4:**
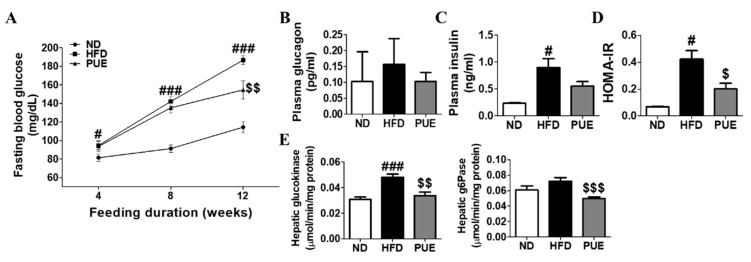
Effect of *P. umbrosa* root extract on glucose metabolism and insulin resistance in DIO mice. (**A**) Fasting blood glucose; (**B**) Plasma glucagon; (**C**) Plasma insulin; (**D**) HOMA-IR; (**E**) Hepatic glucogenic enzyme activity. (**A**–**E**) Data are presented as means ± SEM.; ND, normal diet (AIN-93G); HFD, high-fat diet (60 kcal% fat); PUE, HFD + *P. umbrosa* root extract (1% *w*/*w*). Significant differences between HFD and ND are indicated by ^#^ *p* < 0.05; ^###^ *p* < 0.001. Significant differences between HFD and PUE are indicated by ^$^ *p* < 0.05; ^$$^ *p* < 0.01; ^$$$^ *p* < 0.001. HOMA-IR, homeostasis model assessment of insulin resistance; G6Pase, glucose-6-phosphatase.

Fasting blood glucose (FBG) levels were markedly higher in the HFD group than in the ND group at weeks 4, 8, and 12. PUE supplementation significantly decreased FBG levels compared to those in the HFD group at week 12 (Figure 4A). The HOMA-IR was significantly lowered by PUE, indicating a decrease in insulin resistance (Figure 4D). Hepatic glucokinase (GK) and G6Pase activities were significantly decreased in the PUE group compared to those in the HFD group (Figure 4E).

### 2.6. PUE Ameliorated Hepatic Steatosis as a Modulator of Hepatic Lipid Metabolism 

**Figure 5:**
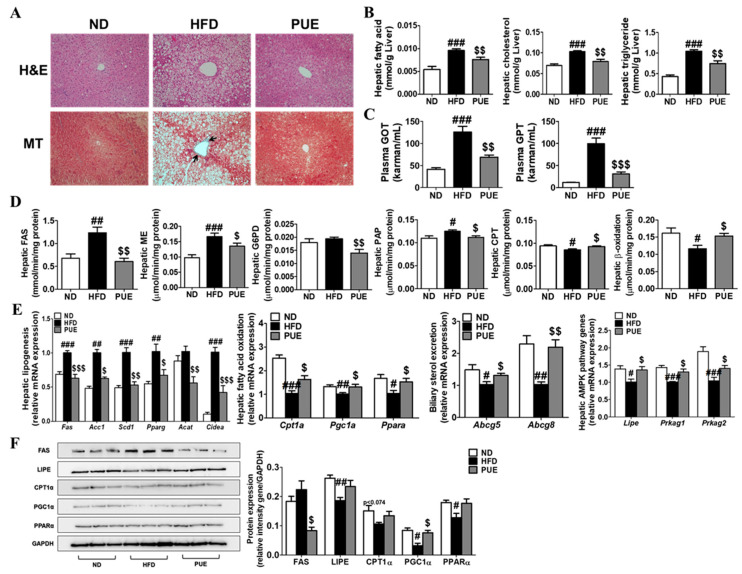
Effect of *P. umbrosa* root extract on hepatic lipids. (**A**) Hepatic morphology (×200); (**B**) Levels of hepatic lipids; (**C**) Hepatotoxicity markers; (**D**) Hepatic lipid-regulating enzyme activities; (**E**) Hepatic gene expression; (**F**) Western blot analysis of FAS, LIPE, PGCLA, CPT1A*,* and PPARA expression. Data are presented as means ± SEM. (**A**) Arrow indicates fibrosis-positive staining (blue). (**B**–**F**) Data are presented as means ± SEM.; ND, normal diet (AIN-93G); HFD (60 kcal% fat); PUE, HFD + *P. umbrosa* root extract (1% *w*/*w*). Significant differences between HFD and ND are indicated: ^#^ *p* < 0.05; ^##^ *p* < 0.01; ^###^ *p* < 0.001. Significant differences between HFD and PUE are indicated by ^$^ *p* < 0.05; ^$$^ *p* < 0.01; ^$$$^ *p* < 0.001. H&E, hematoxylin and eosin staining; MT, Masson’s trichrome staining; GOT, glutamic oxaloacetic transaminase; GPT, glutamic pyruvate transaminase; FAS, fatty acid synthase; ME, malic enzyme; G6PD, glucose-6-phosphate dehydrogenase; PAP, phosphatidate phosphohydrolase; CPT, carnitine palmitoyl transferase.

The HFD group had larger and more numerous hepatic lipid droplets compared to the ND group. In contrast, PUE supplementation reduced the number and size of lipid droplets. MT staining of the liver tissue revealed a blue-stained fibrotic area in the HFD group, whereas this was not observed in the ND and PUE groups (Figure 5A). Hepatic FA, CHOL, and TG levels were significantly higher in the HFD group than in the ND group (*p* < 0.001). In the PUE group, there were remarkable decreases in hepatic FA, CHOL, and TG levels compared to those in the HFD group (*p* < 0.01) (Figure 5B). In addition, levels of hepatotoxicity markers, such as plasma GOT and GPT levels, were markedly increased in the HFD group compared to the ND group, whereas there was a significant reduction in the PUE group compared to the HFD group (Figure 5C). PUE supplementation markedly attenuated hepatic lipogenic enzyme activities (FAS, ME, G6PD, and PAP) and the expression of lipogenesis-related genes (*Fas, Acc1, Scd1, Pparg*, *Acat*, and *Cidea*) and increased hepatic FAO enzyme activities (CPT and beta-oxidation) and the expression of FAO-related genes (*Cpt1a, Pgc1a*, and *Ppara*) compared to the HFD group (Figure 5D,E). The expression of biliary sterol excretion-related genes (*Abcg5* and *Abcg8*) and AMPK signaling pathway genes (*Lipe, Prkag1*, and *Prkag2*) in the PUE group was significantly higher than that in the HFD group (Figure 5E). Western blot analysis of the liver showed that FAS protein expression, a hepatic lipogenic factor, was decreased in PUE-treated mice when compared with HFD-fed mice (Figure 5F).

### 2.7. PUE Promoted BAT Activation and Browning of subWAT

**Figure 6:**
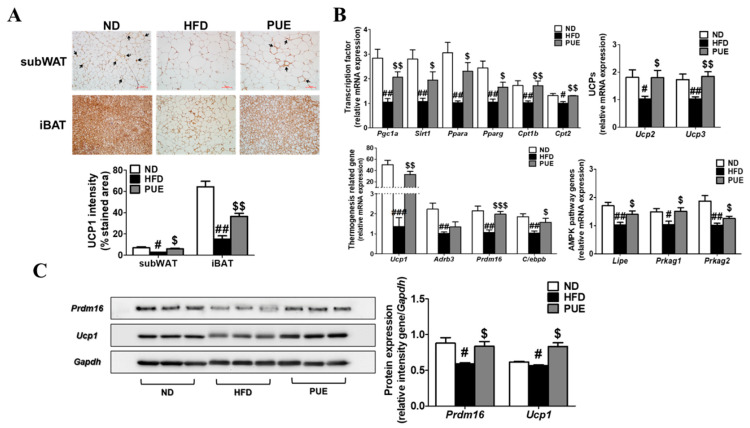
Effect of *P. umbrosa* root extract on activating iBAT and promoting subWAT. (**A**) Immunostaining of UCP1 by IHC (×200) and UCP1 intensity (%); (**B**) Browning-related gene expression in subWAT; (**C**) Western blot analysis of PRDM16 and UCP1 expression in iBAT. (**A**) Arrow indicates UCP1-positive staining. (**A**–**C**) Data are presented as means ± SEM.; ND, normal diet (AIN-93G); HFD, high-fat diet (60 kcal% fat); PUE, HFD + *P. umbrosa* root extract (1% *w*/*w*). Significant differences between HFD and ND are indicated by ^#^ *p* < 0.05; ^##^ *p* < 0.01; ^###^ *p* < 0.001. Significant differences between HFD and PUE are indicated by ^$^ *p* < 0.05; ^$$^ *p* < 0.01; ^$$$^ *p* < 0.001. IHC, immunohistochemistry.

IHC staining of subWAT and iBAT was performed using an anti-UCP1 antibody (Figure 6A). UCP1-IHC of subWAT and IBAT revealed a smaller multilocular lipid droplet accumulation and significantly increased UCP1 expression in PUE mice compared with HFD-fed mice (Figure 6A). Furthermore, the levels of transcription factors (*Pgc1a, Sirt1, Ppara, Pparg, Cpt1b,* and *Cpt2*) and browning markers (*Ucp1, Prdm16,* and *C/ebpb*) in the subWAT of the PUE group were remarkably higher than those in the HFD group. Moreover, in the subWAT, PUE treatment resulted in a significant upregulation of UCP genes (*Ucp2* and *Ucp3*) and AMPK pathway genes (*Lipe, Prkag1*, and *Prkag2*) compared to the HFD group (Figure 6B). In iBAT, the protein expression of PRDM16 and UCP1, both browning markers, was markedly lower in the HFD group than in the ND cohort, whereas that of the PUE-treated group was significantly higher than that in the HFD group (Figure 6C). Therefore, we concluded that PUE treatment promoted BAT activation and WAT browning.

## 3. Discussion

Obesity, a state of excessive accumulation of body fat, causes insulin resistance, T2DM, NAFLD, and cardiovascular disease. The roots and leaves of *P. umbrosa* can be used as food raw materials and are used for pain relief and anti-inflammatory allergic diseases in traditional herbal medicine [16,17]. *Dipsacus asperoides* (*D. asperoides*), similar in appearance to *P. umbrosa*, is known to be effective in improving osteoporosis, fracture healing and proliferation and differentiation of osteoblasts. These two species are often misused or mixed because they are morphologically similar and have a similar name, ‘Sok-dan’, in Korea [18]. The marker compounds of *D. asperoides* and *P. umbrosa* are different; they can be distinguished through marker compound analysis (*D. asperoides*: loganin, sweroside, and akebia saponin D; *P. umbrosa*: shanzhiside methyl ester) [19]. The content of shazhiside methyl ester in the extract of P. umbrosa used in this experiment was 0.72%, which significantly exceeded the standard of 0.007% for the content of indicator substances by the South Korea Ministry of Food and Drug Safety (Supplementary Figure S1). Meanwhile, the anti-obesity effects of *P. umbrosa* have not yet been clarified. Therefore, in the current study, we evaluated the therapeutic efficacy and underlying molecular mechanisms of PUE on obesity and its comorbidities through in vitro and in vivo tests. 

Our experiments showed similarities between gene expression patterns in the thermogenesis of adipose tissue from 3T3-L1 adipocytes and HFD-fed mice. PUE treatment diminished HFD-induced obesity, hepatic steatosis, and insulin resistance and increased thermogenesis and browning responses. The change in body weight gain during the experimental period was significantly lower in the PUE group than in the HFD group, despite the lack of difference in energy intake between the two groups. These results show that the food efficiency ratio was significantly lower in PUE-treated mice. As a result of Pearson’s correlation analysis, there was a significantly positive correlation in the HFD group than in the PUE group between HOMA-IR and BWG (g/day). In addition, the HFD group showed a positive correlation between HOMA-IR and the perirenal, mesenteric, and subcutaneous WAT. In particular, the mesenteric WAT showed a significant difference in the HFD group (Supplementary Table S1A). In addition, PUE supplementation regulated energy metabolism by increasing energy expenditure. PPARA is highly expressed in the small intestine of both mice and humans. PPARA expression is important in intestinal lipid metabolism, in which the catabolism of fatty acids, storage, transfer through the enterocyte, and secretion as triacylglycerols take place [20]. In the small intestine, the decrease in *Ppara,* cluster of differentiation 36 (*Cd36*)*,* fatty acid transport protein 4 (*Fatp4*), and *Apob48* expression increased lipid excretion in the feces [21]. In the present study, PUE treatment regulated genes involved in lipid absorption in the small intestine, thereby increasing the excretion of lipids through feces. 

AMPK is an important molecule in the regulation of cellular energy balance [22]. AMPK activation stimulates lipolysis through phosphorylation of HSL and increases the secretion of adiponectin, resulting in augmented ATP generation and oxidation of fatty acids and glucose in the liver and adipose tissues [23]. The PUE group showed a significantly increased expression of thermogenesis-related (*Ucp1*) and AMPK-related genes, such as *Lipe* (*HSL*), compared to the HFD group. In addition, the number and size of lipid droplets were remarkably decreased in the PUE group compared to those in the HFD group, as determined by morphological observations of adipose tissue. Thus, PUE induced an increased energy expenditure, thereby inhibiting weight gain and fat accumulation in HFD-fed rodents. Adipose tissue is known to express and secrete a variety of hormones and cytokines, such as leptin, resistin, and adiponectin, which are generally referred to as adipocytokines [24]. In this study, the plasma leptin, resistin, and the L:A ratio values in the PUE group were significantly increased compared to those in the HFD group. In contrast, adiponectin levels were significantly increased in PUE-treated mice. Supplementation with PUE significantly decreased plasma TC, PL, and non-HDL-C levels compared to those in the HFD group. Adiponectin has a beneficial effect on lipid metabolism through the activation of AMPK in the liver and skeletal muscles and serves as an adipokine with vascular protective effects [25]. PUE supplementation of HFD decreased hepatic and WAT lipid droplet accumulation, as well as intracellular lipid accumulation in vitro. In addition, PUE treatment improved HFD-induced collagen accumulation in the liver and WAT. Overall, PUE supplementation ameliorated plasma lipid and adipokine levels, resulting in decreased fibrosis in the liver and WAT.

Previous studies have reported that long-term overexpression of hepatic GK results in increased hepatic TG content, which leads to insulin resistance and T2DM [26]. Hepatic gluconeogenesis can be controlled by decreasing the activity of G6Pase and phosphoenolphosphate carboxykinase (PEPCK), which contributes to improvements in hepatic insulin sensitivity [27]. In the present study, PUE treatment significantly reduced plasma FBG levels according to feeding duration. The expression of AMPK pathway-related genes was significantly upregulated by PUE supplementation in WAT and remarkably decreased hepatic GK and G6Pase activity compared to the HFD group. 

Obesity, hepatic steatosis, and dyslipidemia are also important for the release of body lipids. In the case of dietary fats, some are not stored in the body and are excreted out of the body because of alterations in lipid metabolic enzyme activities and/or gene expression in the small intestine with consequent enhanced fecal lipid excretion [28]. Hepatic steatosis is the biochemical result of an imbalance between complex pathways involved in lipid metabolism; therefore, NAFLD is associated with an increased risk of developing various metabolic diseases, such as dyslipidemia and insulin resistance [21]. Mottillo et al. suggested that the AMPK pathway in adipocytes is important for regulating lipolysis [29]. PUE decreases lipid concentrations and hepatic lipogenesis activities, such as fatty acid and triglyceride synthesis. In addition, PUE treatment further decreased protein and gene expression in hepatic lipogenesis, which was increased by the adaptive response to HFD. In addition, the expression of genes related to the FAO and AMPK pathways in the liver was higher in the PUE group than in the HFD group. PUE markedly decreased hepatic lipid levels and increased hepatic *Abcg5* and *Abcg8* mRNA expression, suggesting that PUE treatment inhibited the hepatic lipid load by promoting biliary sterol excretion. We observed that PUE supplementation improved HFD-induced collagen accumulation in the liver and WAT. Moreover, decreased plasma GOT and GPT levels were observed in PUE-treated mice, indicative of reduced liver damage otherwise induced by HFD. In addition, the HFD group showed a significant positive correlation between liver weight and the plasma GOT concentration. However, there was not a significant correlation between the liver weight and the plasma GOT level in PUE mice (Supplementary Table S1B). Therefore, our data indicate that PUE treatment may contribute to the suppression or improvement of DIO and obesity-associated metabolic disorders, particularly hepatic steatosis.

Brown fat is an adipose tissue characterized by increased heat release and energy expenditure due to the presence of numerous mitochondria [30]. BAT is a specialized tissue that exhausts energy in the form of heat by uncoupling FAO from ATP production via UCP1 [8,31]. In the present study, to determine whether PUE induces subWAT browning and iBAT activation, IHC (UCP1) staining was performed. As a result, HFD-fed mice produced large lipid droplets with diminished IHC staining of UCP1. However, we found that subWAT browning and iBAT activation by PUE supplementation increased UCP1 protein expression and upregulated browning-related gene expression. A previous study indicated that due to the energy expenditure function of thermogenic adipose tissue, BAT activation may be a potential target for obesity or metabolic disease treatment by regulating energy balance [32]. Our results indicate that PUE treatment significantly increased the expression of browning markers (*Pgc1a, Ucp1*, and *Prdm16*) in 3T3-L1 adipocytes and subWAT. In addition, the expression of AMPK pathway-related genes (*Prkag1, Prkag2*, and *Lipe*) in subWAT was significantly increased by PUE treatment in HFD-fed mice, together with an increased expression of genes associated with transcription regulators (*Adrb3, Cpt1b, Cpt2, Sirt1, Ppara*, and *Pparg*). Importantly, PUE treatment not only activated Ucp1 protein expression in subWAT and iBAT, but also increased energy expenditure as measured via an indirect calorimeter. In subWAT, the mRNA expression of *Ucp1*, the browning marker, showed a negative correlation with BWG (g/day), which was more significant in the PUE group than in the HFD group (Supplementary Table S1C). Taken together, PUE exerts an anti-obesity effect through not only increased energy expenditure, but also the amelioration of lipid and glucose metabolism. Thus, our study indicates that PUE is a potential therapeutic candidate for the treatment of obesity and its complications by playing various roles in the induction of white adipose browning, activation of brown adipocytes, promotion of lipid catabolism, and lipid excretion.

In conclusion, the present study indicates that PUE treatment can reduce diet-induced obesity and modulate obesity-associated metabolic disorders, including insulin resistance, hepatic steatosis, and inflammation. First, PUE exhibited anti-obesity effects by increasing the expression of genes involved in thermogenesis via the regulation of lipid metabolism in WAT, which increases energy expenditure. In the liver, PUE prevented hepatic steatosis by increasing oxidative metabolism while elevating biliary sterol excretion and suppressing the expression of lipogenic genes. In addition, PUE supplementation increased energy expenditure and regulated fecal lipid excretion, leading to reduced body weight gain. In particular, PUE remarkably activated browning of subWAT via upregulation of UCP1 protein levels and browning-related gene expression and promoted iBAT activation via augmented UCP1 and PRDM16 protein expression. Taken together, the present findings suggest that PUE represents a potential candidate for the prevention or treatment of obesity and related metabolic disorders.

## 4. Materials and Methods

### 4.1. Preparation of P. umbrosa Root Ethanol Extract

*Phlomisumbrosa* root was provided by PUREHERB Co., Ltd. (Andong, Korea). PUE was extracted with 70% ethanol by sonication at 40 °C, repeated twice for 5 h. The extract was collected, concentrated under reduced pressure, and then freeze-dried (yield = 20.5%). The extract was found to contain 7.2 mg/mL of shanzhiside methyl ester, the principle effective iridoid glycoside from PUE, through high-performance liquid chromatography (HPLC). 

### 4.2. Cell Culture and Cell Chemical Staining

#### 4.2.1. Cell Culture and Treatment

3T3-L1 pre-adipocytes (ATCC, Manassas, VA, USA) were grown in Dulbecco’s modified Eagle’s medium (DMEM; Nissui Pharmaceutical, Tokyo, Japan) supplemented with 10% (*v/v*) newborn calf serum and 1% (*v/v*) penicillin–streptomycin solution as a mixed antibiotic at 37 °C in a humidified 5% CO_2_ atmosphere [33]. For adipocyte differentiation, 3T3-L1 preadipocyte cells were cultured for two days to post-confluence and then stimulated for two days with differentiation medium (DMEM containing 10% fetal bovine serum, 0.5 mM 3-isobutyl-1-methylxanthine, 1 µM dexamethasone, and 1 µg/mL insulin). Subsequently, the cells were incubated for eight days in DMEM containing 10% fetal bovine serum and 10 µg/mL insulin. 3T3-L1 pre-adipocytes were treated with or without PUE (100 µg/mL) during differentiation. After day 8, samples were harvested, and RNA was extracted from the samples using TRIzol reagent (Invitrogen Life Technologies, Grand Island, NY, USA) according to the manufacturer’s instructions.

#### 4.2.2. Oil Red O Staining

Oil Red O staining after 8 and 12 days of adipocyte differentiation in 3T3-L1 cells treated with the differentiation drugs methylisobutylxanthine, dexamethasone, and insulin (MDI) and PUE (100 μg/mL). Differentiated 3T3-L1 adipocytes were fixed in 10% formalin in phosphate-buffered saline for 1 h and washed twice with 60% isopropanol. The fixed cells were then stained with Oil Red O solution for 30 min and washed with distilled water. After drying, fixed cells were imaged under a microscope. The Oil Red O solution taken up by the cells was then extracted using 100% isopropanol, and its optical density was measured at 520 nm.

### 4.3. Animal Experimental Design and Diet

C57BL/6J mice (four-week-old, male, n = 30) were obtained from Jackson Laboratories (Bar Harbor, ME, USA). All mice were individually housed under a 12 h light/dark cycle at a controlled temperature (22 ± 2 °C). The mice were fed a normal chow diet for 1 week after arrival. After adaptation, the mice were randomly divided into three groups and fed a normal diet (ND, AIN-93G, n = 10), high-fat diet (HFD, n = 10, 60 kcal% fat), or HFD + 1% PUE (*w/w*, n =10). Mice had free access to food and water during the experimental period. At the end of the experimental period, the mice were anesthetized with isoflurane after 12 h of fasting. Blood was collected from the inferior vena cava to determine plasma lipid, adipokine, and hormone concentrations. The liver, muscle, and adipose tissues were removed, rinsed with physiological saline, weighed, immediately frozen in liquid nitrogen, and stored at −70 °C until analysis. Animal studies were performed using protocols approved by the Kyungpook National University Industry Foundation (Approval No. 2017-0109).

### 4.4. Measurements of Energy Expenditure 

Energy expenditure was measured using an indirect calorimeter (Oxylet, Panlab, Cornella, Spain). The mice were placed in individual metabolic chambers at 25 °C with free access to food and water. O_2_ and CO_2_ analyzers were calibrated using high-purity gases. O_2_ consumption (VO_2_) and CO_2_ production (VCO_2_) were recorded at 3 min intervals using computer-assisted data acquisition software (Chart 5.2; AD Instrument, Sydney, Australia) over a 24 h period, and the data were averaged for each mouse. Energy expenditure (EE) was calculated using the following formula [34]:

EE (kcal·day^−1^·bodyweight^−0.75^) = VO_2_ × 1.44 × [3.815 + (1.232 × VCO_2_/VO_2_)

### 4.5. Morphology of Liver and Adipose Tissues

The liver, epiWAT, subWAT, and interscapular BAT (iBAT) were removed from each mouse and fixed in a buffer solution of 10% formalin. Fixed tissues were routinely processed for paraffin embedding, and 4 µm sections were prepared and stained with hematoxylin and eosin (H&E) and Masson’s trichrome (MT). For immunohistochemistry (IHC) staining of subWAT and iBAT, a poly anti-Ucp1 antibody (Abcam, ab234430, Carlsbad, CA, USA) at a dilution of 1:50 was used. All stained areas were viewed using an optical microscope (Zeiss Axioscope, Jena, Germany) at a magnification of 200×.

### 4.6. Biochemical Parameters of Plasma, Hepatic, and Fecal Lipids

The concentrations of plasma TG, total cholesterol (TC), and high-density lipoprotein cholesterol (HDL-C) were measured using a commercial kit (Asan Pharm Co., Seoul, Korea). Apolipoproteins A-1 and B (Apo A-1 and Apo B) levels were measured using enzymatic kits (Eiken, Japan). Plasma FFA and phospholipid (PL) levels were measured using the Wako enzymatic kit (Wako Chemicals, Richmond, VA, USA). The value of non-HDL-C was calculated as follows: non-HDL-C = (TC) − (HDL-C). The HDL-C to TC ratio (HTR) was calculated as follows: HTR (%) = (HDL-C)/(TC) × 100. 

The atherogenic index (AI) was calculated as follows: AI = [(TC) − (HDL-C)]/(HDL-C). Hepatic and fecal lipid contents were extracted [35], and dried lipid residues were dissolved in 1 mL of ethanol. Triton X-100 (Sigma-Aldrich, St. Louis, MO, USA) and a sodium cholate solution in distilled water were added to 200 µL of the dissolved lipid solution for emulsification. TG, cholesterol (CHOL), and fatty acid (FA) concentrations were analyzed using the same enzymatic kit used for plasma analyses.

### 4.7. Determination of Hormones and Adipokines in Plasma

Levels of plasma hormones (insulin and glucagon) and adipokines (leptin and resistin) were determined using the MILLIPLEX^®^ MAP mouse metabolic magnetic bead panel (Merck, Temecula, CA, USA). Plasma adiponectin was quantified using mouse adiponectin/Acrp30 (R&D Systems ™, Bio-Techne, Minneapolis, MN, USA).

### 4.8. Fasting Blood Glucose and Insulin Resistance Index

Fasting blood glucose (FBG) levels were determined from tail vein blood samples after fasting for 12 h using a glucose analyzer (One Touch Select Plus, Lifescan Inc., Malvern, PA, USA). A homeostatic model assessing insulin resistance (HOMA-IR) was calculated according to the following formula [36]:

HOMA-IR = [FBG (mmol/L) × fasting insulin (µU/mL)]/22.5.

### 4.9. Plasma Glutamic Oxaloacetic Transaminase (GOT) and Glutamic Pyruvic Transaminase (GPT) Activities

GOT and GPT activities were determined using a commercially available kit (Asan Pharm Co., Seoul, Korea).

### 4.10. Determination of Hepatic Enzyme Activity

A hepatic enzyme source was prepared according to the method developed by Hulcher and Oleson [37] with slight modifications. Fatty acid synthase (FAS) activity was determined via a spectrophotometric assay according to the method of Nepokroeff et al. [38]. Malic enzyme (ME) activity was measured using the method described by Ochoa et al. [39]. Glucose-6-phosphate dehydrogenase (G6PD) activity was determined as described by Pitkänen et al. [40]. Phosphatidate phosphohydrolase (PAP) activity was measured using the method described by Walton and Possmayer [41]. Carnitine palmitoyl transferase (CPT) activity was determined according to Markwell et al. [42]. Fatty acid β-oxidation was measured by monitoring the reduction of NAD to NADH in the presence of palmitoyl-CoA, as described by Lazarow et al. [43]. Glucokinase (GK) activity was determined using a continuous spectrometric assay as described by Newgard et al. [44]. Glucose-6-phosphatase (G6Pase) activity was determined using the method described by Alegre et al. [45], with slight modifications. 

### 4.11. Real-Time qPCR Analysis

Total RNA was extracted using TRIzol reagent (Invitrogen Life Technologies, Carlsbad, CA, USA), according to the manufacturer’s instructions. Total RNA (1 μg) was reverse-transcribed into cDNA using the Takara^®^ Prime Script™ Real Time reagent kit (Takara, Shiga, Japan), and mRNA expression was quantified by RT-qPCR using a SYBR green PCR kit (Enzynomics, Daejeon, Korea) and the CFX96TM real-time PCR system (Bio-Rad, Hercules, CA, USA). Gene-specific mouse primers were used as indicated in Table 2. The cycle threshold (C_t_) data were normalized to that of *GAPDH*, and relative gene expression was calculated using the 2^−^^ΔΔCt^ method [46]. 

### 4.12. Western Blotting

Liver and iBAT proteins were extracted with tissue protein extraction reagent (TPER) buffer (Thermo Fisher Scientific, Rockford, IL, USA) containing protease and phosphatase inhibitor cocktails. Extracted proteins were quantified using the Bradford method and subjected to sodium dodecyl sulfate-polyacrylamide gel electrophoresis (SDS-PAGE) on 10% SDS polyacrylamide gels. Western blotting was performed after the samples were transferred to polyvinylidene fluoride membranes (Merck Millipore, Billerica, MA, USA). The membrane was blocked with Tris-buffered saline in 0.1% tween-20 (TBST) containing 5% bovine serum albumin (BSA) or 5% skim milk for 1 h and incubated with rabbit anti-FAS (1:1000; Cell Signaling, #3180), anti-LIPE (lipase E, hormone-sensitive lipase, Hsl) (1:1000; Abcam, ab45422), anti-PGC1A (peroxisome proliferator-activated receptor gamma coactivator 1-alpha) (1:1000; Abcam, ab191838), anti-PPARA (Peroxisome proliferator-activated receptor alpha) (1:1000; Abcam, ab24509), anti-UCP1 (1:1000; Abcam, ab234430), anti-PRDM16 (PR domain containing 16) (1:1000; Abcam, ab106410), and anti-CPT1A (Carnitine palmitoyl-CoA transferase 1-alpha) (1:1000; Abcam, ab128568), respectively. As a loading control, glyceraldehyde-3-phosphate dehydrogenase (GAPDH) was detected using primary mouse anti-GAPDH (1:1000; Santa Cruz Biotechnology, sc-32233). Immunoreactive antigens were then recognized using horseradish peroxidase (HRP)-conjugated rabbit anti-IgG (1:3000; Cell Signaling, #7074S) or mouse anti-IgG (1:3000; Cell Signaling, #7076S). Protein bands were visualized using Westar Eta C Ultra 2.0, (Cyanagen, Bologna, Italy). Immunoreactive bands were quantified using Image J software (Image J version 1.8, NIH, Bethesda, MD, USA)).

### 4.13. Statistical Analysis

Data are expressed as means ± standard error of the mean (SEM). All statistical analyses were performed using Statistical Package for Social Sciences (SPSS) software (v. 23.0; SPSS Inc., Chicago, IL, USA) for Windows. Statistical significance was determined using unpaired Student’s *t*-tests to compare two groups, as indicated in the Figure legends. Statistically significant differences between means were defined as *p* < 0.05.

## Figures and Tables

**Table 1:** Effect of 12 weeks of *Phlomis umbrosa* root extract treatment on plasma lipid profiles in DIO mice.

	ND	HFD	PUE
TG (mmol/L)	1.09 ± 0.06	1.35 ± 0.06 ^##^	1.25 ± 0.11
TC (mmol/L)	3.70 ± 0.14	5.84 ± 0.17 ^###^	5.16 ± 0.22 ^$^
PL (mg/dL)	6.50 ± 1.67	33.55 ± 2.24 ^###^	20.68 ± 2.39 ^$$^
HDL-C (mmol/L)	0.77 ± 0.04	1.13 ± 0.04 ^###^	1.06 ± 0.03
Non-HDL-C (mmol/L)	2.93 ± 0.13	4.71 ± 0.14 ^###^	4.10 ± 0.18 ^$^
HTR (%)	20.96 ± 1.19	19.73 ± 1.09	21.27 ± 1.28
AI	3.89 ± 0.26	4.20 ± 0.33	3.82 ± 0.30
Apo A-1 (mg/dL)	83.02 ± 1.16	78.65 ± 0.64 ^#^	81.02 ± 1.03
Apo B (mg/dL)	6.88 ± 0.39	10.29 ± 1.96	5.82 ± 0.69
Apo A-1/Apo B	12.06 ± 0.59	7.64 ± 1.31 ^#^	13.92 ± 1.78 ^$^

Data are presented as means ± SEM; ND, normal diet (AIN-93G); HFD, high-fat diet (60 kcal% fat); PUE, HFD + *P. umbrosa* root extract (1% *w*/*w*). Significant differences between HFD and ND groups are indicated by ^#^ *p* < 0.05; ^##^ *p* < 0.01; ^###^ *p* < 0.001. Significant differences between the HFD and PUE groups are indicated by ^$^ *p* < 0.05, and ^$$^ *p* < 0.01. DIO, diet-induced obesity; FFA, free fatty acid; TG, triglyceride; TC, total cholesterol; PL, phospholipid; HDL-C, high-density lipoprotein cholesterol; non-HDL-C = (TC) − (HDL-C); HTR = [(HDL-C)/(TC)] × 100; AI, atherogenic index = [(TC) − (HDL-C)]/(HDL-C); Apo A-1, apolipoprotein A-1; and Apo B, apolipoprotein B.

**Table 2:** Primer sequences used for RT-qPCR.

Primer	Primer Direction	Sequence
*Gapdh*: Glyceraldehyde-3-phosphate dehydrogenase	Forward Reverse	5′-AAGGTCATCCCAGAGCTGAA-3′ 5′-CTGCTTCACCACCTTCTTGA-3′
*Abcg5*: ATP-binding cassette sub-family G member 5	Forward Reverse	5′-AGAGGGCCTCACATCAACAGA-3′ 5′-CTGACGCTGTAGGACACATGC-3′
*Abcg8*: ATP-binding cassette sub-family G member 8	Forward Reverse	5′-TGGTCAGTCCAACACTCTGG-3′ 5′-ACTGGGTTGCCCATTTATCC-3′
*Acat*: Acetyl-coenzyme A acetyltransferase	Forward Reverse	5′-AGAAATCAAGCAAAGGTCCA-3′ 5′-AGGAGTCCTTGGGTAGTTGT-3′
*Acc1*: Acetyl-CoA carboxylase 1	Forward Reverse	5′-GCCTCTTCCTGACAAACGAG-3′ 5′-TGACTGCCGAAACATCTCTG-3′
*Adrb3*: Adrenergic receptor beta 3	Forward Reverse	5′-ACCAACGTGTTCGTGACT-3′ 5′-ACAGCTAGGTAGCGGTCC-3′
*Apob48:* Apolipoprotein B 48	Forward Reverse	5′-TGGCTCTGATCCCAAATCCCT-3′ 5′-CCGTGCATTCATTGTCGATCT-3′
*Cidea*: Cell death-inducing DNA fragmentation factor alpha subunit-like effector A	Forward Reverse	5′-TTTCAAACCATGACCGAAGTAGCC-3′ 5′-CCTCCAGCACCAGCGTAACC-3′
*Cpt1a*: Carnitine palmitoyl-CoA transferase 1-alpha	Forward Reverse	5′-ATCTGGATGGCTATGGTCAAGGTC-3′ 5′-GTGCTGTCATGCGTTGGAAGTC-3′
*Cpt1b*: Carnitine palmitoyl-CoA transferase 1-beta	Forward Reverse	5′-TGCCTTTACATCGTCTCCAA-3 5′-AGACCCCGTAGCCATCATC-3′
*Cpt2*: Carnitine palmitoyl-CoA transferase 2	Forward Reverse	5′-CAACTCGTATACCCAAACCCAGTC-3′ 5′-GTTCCCATCTTGATCGAGGACATC-3′
*C/ebpb*: CCAAT/enhancer-binding protein beta	Forward Reverse	5′-GGAGACGCAGCACAAGGT-3′ 5′-AGCTGCTTGAACAAGTTCCG-3′
*Fas*: Fatty acid synthase	Forward Reverse	5′-GCTGCGGAAACTTCAGGAAAT-3′ 5′-AGAGACGTGTCACTCCTGGACTT-3′
*Lipe*: Lipase E	Forward Reverse	5′-GGCTCACAGTTACCATCTCACC-3′ 5′-GAGTACCTTGCTGTCCTGTCC-3′
*Pgc1a*: Peroxisome proliferator-activated receptor gamma coactivator 1-alpha	Forward Reverse	5′-AAGTGTGGAACTCTCTGGAACTG-3′ 5′-GGGTTATCTTGGTTGGCTTTATG-3′
*Ppara*: Peroxisome proliferator-activated receptor alpha	Forward Reverse	5′-CCTGAACATCGAGTGTCGAATAT-3′ 5′-GGTCTTCTTCTGAATCTTGCAGCT-3′
*Pparg*: Peroxisome proliferator-activated receptor gamma	Forward Reverse	5′-GCATGGTGCCTTCGCTGA-3′ 5′-TGGCATCTCTGTGTCAACCATG-3′
*Prdm16*: PR domain containing 16	Forward Reverse	5′-CAGCACGGTGAAGCCATTC-3′ 5′-GCGTGCATGCGCTTGTG-3′
*Prkag1*: Protein kinase AMP-activated non-catalytic subunit gamma 1	Forward Reverse	5′-TCTCCGCCTTACCTGTAGTGGA-3′ 5′-GCAGGGCTTTTGTCACAGACAC-3′
*Prkag2*: Protein kinase AMP-activated non-catalytic subunit gamma 2	Forward Reverse	5′-CTCCTCATCCAAAGAGTCTTCGC-3′ 5′-TGGGTGTTGACGGAGAAGAGGA-3′
*Scd1*: Steroly-CoA desaturase 1	Forward Reverse	5′-CCCCTGCGGATCTTCCTTAT-3′ 5′-AGGGTCGGCGTGTGTTTCT-3′
*Sirt1*: Sirtuin 1	Forward Reverse	5′-TGTGAAGTTACTGCAGTGTAA-3′ 5′-GCATAGATACCGTCTCTTGATCTG-3′
*Ucp1*: Uncoupling protein 1	Forward Reverse	5′-ACTGCCACCCCTCCAGTCATT-3′ 5′-CTTTGCCTCACTGAGGATTGG-3′
*Ucp2*: Uncoupling protein 2	Forward Reverse	5′-ACCAAGGGCTCAGAGCATGCA-3′ 5′-TGGCTTTCAGGAGAGTATCTTTG-3′
*Ucp3*: Uncoupling protein 3	Forward Reverse	5′-GGATTTGTGCCCTCCTTTCTG-3′ 5′-AGATTCCCGCAGTACCTGGAC-3′

## Data Availability

Data are contained within the article and supplementary material.

## Supplementary Materials

The following supporting information can be downloaded at: https://www.mdpi.com/article/10.3390/ijms23169295/s1.

## Abbreviations

AMPK	AMP-activated protein kinase
BAT	brown adipose tissue
CPT	carnitine palmitoyltransferase
DIO	diet-induced obesity
EE	energy expenditure
eWAT	epididymal WAT
FAO	fatty acid oxidation
FAS	fatty acid synthase
FER	food efficiency ratio
GOT	glutamic oxaloacetic transaminase
GPT	glutamic pyruvic transaminase
G6PD	glucose-6-phosphate dehydrogenase
HFD	high-fat diet
HOMA-IR	homeostasis model assessment for insulin resistance
ME	malic enzyme
ND	normal diet
NAFLD	non-alcoholic fatty liver disease
PAP	phosphatidate phosphohydrolase
PBS	phosphate buffered saline
PUE	*Phlomis umbrosa* Turcz. root ethanol extract
subWAT	subcutaneous WAT
T2DM	type 2 diabetes mellitus
UCP1	uncoupling protein 1
WAT	white adipose tissue
