# Supplementary material for: Novel Plant Extract Ameliorates Metabolic Disorder through Activation of Brown Adipose Tissue in High-Fat Diet-Induced Obese Mice
Source: Int J Mol Sci. 2022 Aug 18;23(16):9295. doi: 10.3390/ijms23169295 (PMC9409404; doi:10.3390/ijms23169295)
Supplement: Supplementary file 1 [file ijms-23-09295-s001.zip › ijms-1847801-supplementary.pdf]

Article

Novel plant extract ameliorates metabolic disorder through activation of brown adipose tissue in high-fat diet-induced obese mice

Ji-Won Kim,[1#] Young-Mo Yang,[2#] Eun-Young Kwon,[1] and Ji-Young Choi [3*]

[1]Department of Food Sciences and Nutrition, Kyungpook National University, Daegu, Republic of Korea.
[2]Department of Pharmacy, College of Pharmacy, Chosun University, Gwangju, Republic of Korea.
[3]Department of Food and Nutrition, College of Natural Science and Public Health and Safety, Chosun University, Gwangju, Republic of Korea.
[#]These authors contributed equally to this article.

* Correspondence: Prof. Ji-Young Choi, Department of Food and Nutrition, College of Natural Science and Public Health and Safety, Chosun University, 309 Pilmun-daero, Dong-gu, Gwangju, Republic of Korea 61452. Tel.: +82-62-230-7723, Fax: +82-62-225-7726, E-mail: jychoi@chosun.ac.kr

Ⅰ. Supplementary Figures

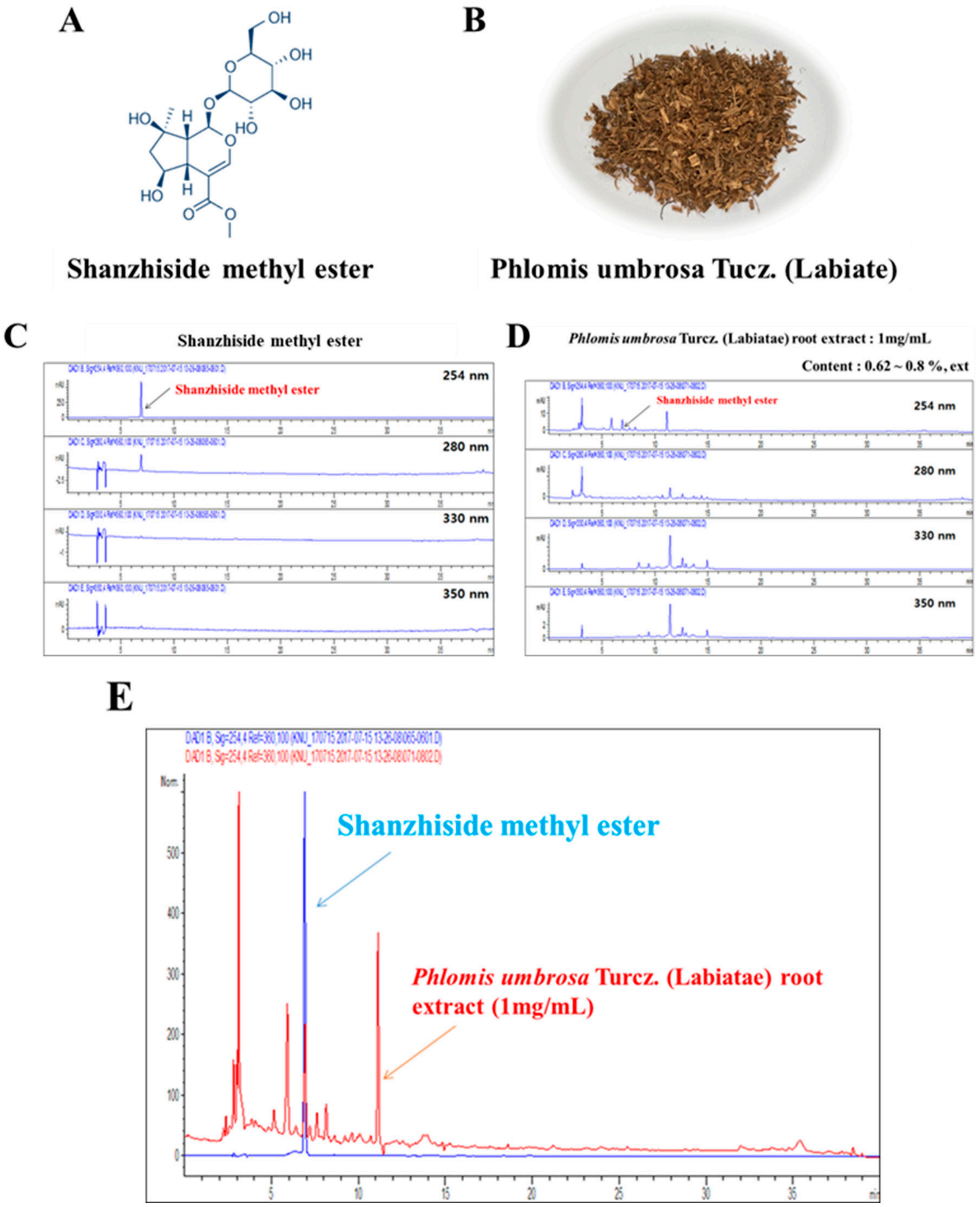

Figure S1. The shanzhiside methyl ester content of *Phlomis umbrosa* Turcz.by HPLC analysis

Ⅱ. Supplementary Tables

Table S1. Pearson correlation analysis in HFD and PUE groups

A

		HOMA-IR	BWG (g/day)	Perirenal WAT (g/ 100g BW)	Mesenteric WAT (g/ 100g BW)	Subcutaneous WAT (g/ 100g BW)
HFD	Pearson Correlation	1	.895*	0.875	.952*	0.570
	Sig. (2-tailed)		0.040	0.321	0.013	0.316
PUE	Pearson Correlation	1	0.334	0.482	0.286	0.101
	Sig. (2-tailed)		0.517	0.333	0.583	0.848

B

		Liver (g/ 100g BW)	Plasma GOT (karman/mL)
HFD	Pearson Correlation	1	.910**
	Sig. (2-tailed)		0.002
PUE	Pearson Correlation	1	-0.132
	Sig. (2-tailed)		0.778

C

		BWG (g/day)	sub_UCP1 (mRNA level)
HFD	Pearson Correlation	1	-0.245
	Sig. (2-tailed)		0.524
PUE	Pearson Correlation	1	-.736*
	Sig. (2-tailed)		0.037

HFD, high-fat diet (60 kcal% fat); PUE, HFD + *P. umbrosa* root extract (1% w/w). HOMA-IR, homeostasis model assessment of insulin resistance; BWG, Body weight gain; WAT, white adipose tissue; GOT, glutamic oxaloacetic transaminase; sub, subcutaneous; *Ucp1*, Uncoupling protein 1
